# Effect of preoperative isolated muscular calf vein thrombosis on the safety and efficacy of open reduction and internal fixation for schatzker type II and III tibial plateau fractures

**DOI:** 10.3389/fsurg.2026.1884502

**Published:** 2026-08-24

**Authors:** Jiliu Huang, Weibo Zhou, Wen Tan, Fulin Zhou, Yaojun Lu

**Affiliations:** 1 Department of Orthopedics, The Second People's Hospital of Changzhou, Changzhou, China; 2 Nanjing Medical University, Nanjing, China

**Keywords:** deep vein thrombosis, isolated muscular calf vein thrombosis, knee function recovery, open reduction and internal fixation, tibial plateau fracture

## Abstract

**Background:**

Orthopedic surgeons may underestimate the thrombotic risks associated with isolated muscular calf vein thrombosis (IMVT) and its influence on postoperative outcomes. This retrospective cohort study aimed to explore the impact of preoperative IMVT on perioperative safety and clinical efficacy of open reduction and internal fixation (ORIF) for Schatzker type II and III tibial plateau fractures.

**Methods:**

Patients with Schatzker type II or III tibial plateau fractures receiving ORIF between January 2021 and March 2025 at our institution were consecutively enrolled. Subjects were divided into the study group (patients with preoperative IMVT) and comparison group (patients without preoperative IMVT). Baseline demographics, injury-related data, intraoperative parameters, postoperative VAS pain scores, knee flexion range of motion, and the occurrence of postoperative deep vein thrombosis (DVT) were collected. All patients completed a minimum 6-month follow-up.

**Results:**

Overall, 242 patients were included. Baseline demographic characteristics, lifestyle factors, laboratory indices, preoperative knee swelling rate and operative duration were comparable between the two groups (all *p* > 0.05). Postoperative DVT developed in 12 of 17 patients (70.6%) in the study group versus 32 of 225 patients (14.2%) in the comparison group; the difference was statistically significant (*p* < 0.05). Among patients with postoperative DVT, non-muscular calf vein thrombosis was identified in 7/12 (58.3%) of the study group and 19/32 (59.4%) of the comparison group. The study group exhibited inferior knee flexion angle at 1 month postoperatively, whereas no intergroup differences in knee flexion were detected at 3 and 6 months after surgery.

**Conclusion:**

Preoperative isolated muscular calf vein thrombosis is associated with a higher risk of postoperative DVT and impaired short-term recovery of knee flexion function in patients undergoing ORIF for tibial plateau fractures.

## Introduction

Tibial plateau fractures (TPF), a common type of lower limb fracture, account for approximately 1% of all human fractures (1, 2), and about 8% of fractures in elderly patients (3). Lateral tibial plateau fractures with depression are the most frequently observed type in clinical practice (4), Open reduction and internal fixation (ORIF) is considered the gold standard for the treatment of displaced tibial plateau fractures (5). The determinants of postoperative satisfaction following tibial plateau fracture surgery are multifactorial. Key factors such as knee range of motion (functional mobility), pain levels, and the occurrence of postoperative complications are all closely linked to patient satisfaction (6–8).

Deep vein thrombosis (DVT) is a common complication following TPF surgery, primarily presenting as pain and swelling in the lower limb. When a DVT dislodges and travels through the bloodstream to the lungs, it can cause pulmonary embolism (PE), a significant factor in unexpected perioperative mortality (9). Therefore, postoperative DVT poses a major threat to patient safety. In recent years, ongoing clinical research has identified an increasing number of risk factors associated with DVT, such as cerebrovascular diseases, respiratory conditions, anesthesia, and the time from injury to surgery (10). Preventive measures targeting these risk factors have significantly reduced the incidence of DVT (11). The increasing application of lower limb Doppler ultrasound has driven higher detection rates of asymptomatic DVT and isolated muscular calf vein thrombosis. This has revealed a clinical dilemma: while some literature links these findings to an increased PE risk in TPF patients (12, 13), other studies contend that isolated muscular calf vein thrombosis does not increase PE risk and warrants only oral anticoagulation, not vena cava filter placement.

The deep veins of the lower limb are anatomically categorized into proximal (central) and distal (peripheral) systems. Clinically, both proximal and distal deep vein thrombosis (DVT) represent major threats to patient morbidity and mortality. Consequently, ultrasound examination for DVT is routinely employed to guide subsequent therapeutic decisions. Muscular veins, such as those within the soleus and gastrocnemius muscles, are part of the distal deep venous system. These vessels are typically of small caliber and drain into the larger distal deep veins—namely, the anterior tibial, posterior tibial, and peroneal veins—which ultimately converge into the proximal deep venous system. Furthermore, most patients with muscular calf vein thrombosis are asymptomatic. Over time, these thrombi are often spontaneously absorbed or become organized into chronic residuals. Whether isolated muscular calf vein thrombosis warrants active treatment remains a subject of ongoing debate, with no established consensus (14–16). However, clinical data regarding tibial plateau fractures combined with preoperative isolated muscular calf vein thrombosis are limited.

The primary endpoint of this study was the incidence of newly developed postoperative deep vein thrombosis (DVT) within 6 months after surgery, which represented the core research objective focusing on venous thrombotic risk associated with preoperative isolated muscular calf vein thrombosis. Secondary endpoints included intraoperative blood loss, postoperative knee flexion range of motion, VAS pain scores at 1, 3 and 6 months after surgery, limb swelling rate at discharge, thrombus subtype distribution among patients with DVT, and other perioperative complications. The study protocol was approved by the local Institutional Review Board (IRB).

### Patients

Inclusion Criteria: Patients were included if they met all of the following: (1) Radiographically confirmed Schatzker type II or III tibial plateau fracture with clear surgical indications; (2) Underwent ORIF surgery after providing full informed consent; (3) Preoperative color Doppler ultrasound of both lower limbs confirmed the presence of isolated muscular calf vein thrombosis (IMVT); (4) Patients without IMVT were enrolled as the control group.

Exclusion Criteria: Patients were excluded for any of the following: (1) Age < 18 years; (2) Contraindications to anticoagulant therapy (e.g., due to renal or hematologic diseases); (3) History of malignancy with prior radiotherapy or chemotherapy; (4) Long-term bedridden status; (5) Major trauma or other surgical procedures within 6 months preceding surgery or during the follow-up period; (6) Body mass index (BMI) > 40 kg/m^2^; (7) Intraoperative non-use of a tourniquet or failure to place a drainage tube; (8) Incomplete clinical data or loss to follow-up.

**Figure 1:**
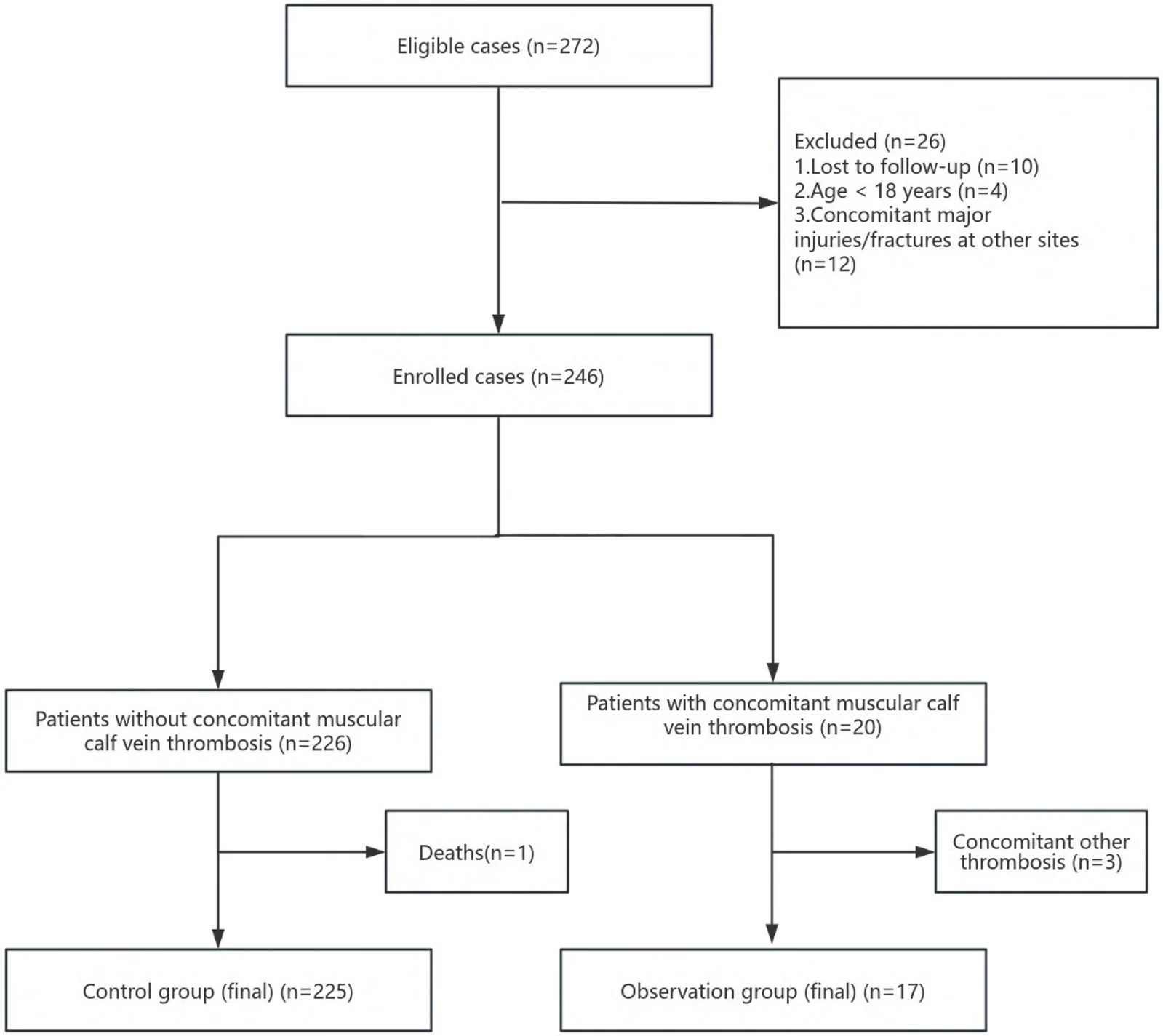
Study flow chart.

**Figure 2:**
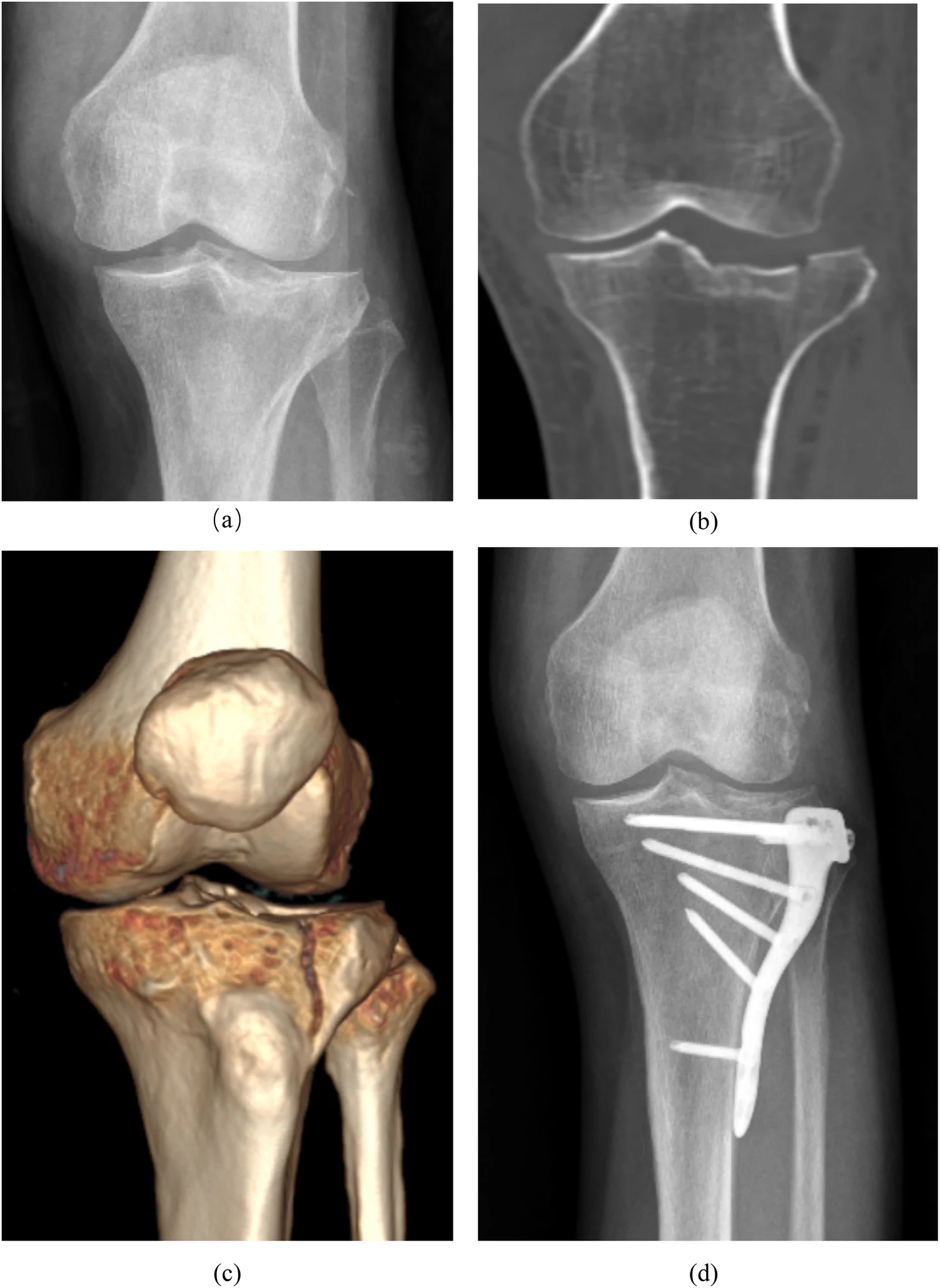
Pre-operative and post-operative imaging of Schatzker type-II tibial plateau fracture. **(a)** Pre-operative anteroposterior radiograph of knee; **(b)** Coronal CT scan showing depressed tibial plateau fracture; **(c)** Three-dimensional CT reconstruction demonstrating fracture morphology; **(d)** Post-operative anteroposterior radiograph after open reduction and internal fixation with lateral rafting plate and screws.

This study was a retrospective trial that ultimately included 242 patients (Figures 1). They were divided into two groups based on the presence or absence of lower extremity isolated muscular calf vein thrombosis (IMVT): the observation group (with IMVT) and the control group (without IMVT). The observation group consisted of 17 patients (7 males and 10 females), while the control group comprised 225 patients (94 males and 131 females). The demographic characteristics and lifestyle habits, including smoking (≥5 cigarettes/day) and alcohol consumption (≥10 g/day), were comparable between the two groups (Table 1). Furthermore, there were no significant differences in comorbidities, preoperative laboratory indices, swelling rate of the tibial plateau, or Visual Analogue Scale (VAS) pain scores (Table 1). Preoperative imaging evidence from x-ray, CT, and three-dimensional CT for all patients confirmed surgical indications (Figures 2A–C).

**Table 1:** Comparison of preoperative demographic and baseline clinical characteristics between the two groups.

Variables	Comparison Group (*n*=225)	Study Group (*n*=17)	Mean difference (95%CI)	Effect size (95%CI)	*p*-value
Age, Years, Mean(SD)	51.4 (15.3)	55.2 (14.9)	−3.73 [−11.32, 3.85]	Cohen's d = 15.31 [−0.74, 0.25]	0.333
Gender					
Male, n(%)	94（41.8）	7（41.2）		0.98 (95%CI:0.36∼2.66)	0.961
Female, n(%)	131（58.2）	10（58.8）			
BMI, kg/㎡，mean（SD）	24.8 (3.4)	24.5 (2.9)	0.31[−1.37, 1.99]	Cohen's d = 3.39[−0.40, 0.58]	0.333
Injured side, n(%)					
Left	121 (53.8)	10 (58.8)		1.23 (95%CI:0.45∼3.34)	0.687
Right,	104 (46.2)	7 (41.2)			
History of smoking and alcohol consumption, n(%)					
Smoking	77	6		1.05 (95%CI:0.37∼2.94)	0.928
Alcohol consumption	80	6		0.989 (95%CI:0.35∼2.77)	0.983
Comorbidities, n (%)					
Hypertension	70 (31.1)	7 (41.2)		1.55 (95%CI:0.57∼4.24)	0.390
Diabetes Mellitus	27 (12.0)	5 (29.4)		3.06 (95%CI:1.00∼9.35)	0.057
Hyperlipidemia	10 (4.4)	2 (11.8)		2.87 (95%CI:0.58∼14.28)	0.202
Coronary Artery Disease	7 (3.2)	2 (11.8)		4.15 (95%CI:0.79∼21.76)	0.125
Preoperative Knee Assessments, mean(SD)					
Swelling Rate (%)	12.0 (4.5)	13.7 (3.7)	−1.66 [−3.85, 0.52]	Cohen's d = 4.41[−0.87， 0.12]	0.135
VAS Pain Score (points)	7.0 (1.1)	7.4 (1.3)	−0.46 [−1.00, 0.09]	Cohen's d = 1.10 [−0.91, 0.08]	0.100
Preoperative Laboratory Findings, mean(SD)					
D-dimer (mg/L)	5.8 (5.6)	8.0 (6.4)	−2.17 [−4.97, 0.63]	Cohen's d = 5.65 [−0.88 0.11]	0.717
Total Cholesterol (mmol/L)	4.3 (1.0)	4.3 (0.8)	0.10 [−0.40, 0.60]	Cohen's d = 1.01[−0.40, 0.59]	0.704

### Surgical technique

All included patients were operated on by an experienced senior attending surgeon and their team. The specific surgical technique was as follows:

1. After the induction of general anesthesia, the patient was placed in the supine position. The surgical field was disinfected three times with povidone-iodine and routinely draped with sterile sheets. Following exsanguination with an Esmarch bandage, the procedure was performed under a pneumatic tourniquet inflated to a pressure of 40 kPa.
2. A lateral approach to the tibial plateau was made. Sequential incisions were made through the skin, subcutaneous fat, and deep fascia. The tibialis anterior muscle was elevated in a limited fashion, the joint capsule was opened, and the lateral plateau was exposed. Upon identifying depression of the lateral plateau, the fragment was elevated and reduced. Any bone defect was grafted with synthetic bone substitute. The reduced fracture fragments were provisionally fixed with Kirschner wires. A rafting plate was positioned on the lateral plateau, and screws were inserted after drilling. Intraoperative fluoroscopy (C-arm) confirmed satisfactory fracture reduction, an even articular surface, and appropriate implant length (Figure 2D). Throughout the procedure, meticulous care was taken to minimize excessive retraction and soft tissue dissection to avoid iatrogenic neurovascular injury.
3. Upon completion, a drainage tube was placed, and the wound was closed in layers. The use of tranexamic acid or other agents affecting coagulation was prohibited. The wound was dressed with cotton padding and an elastic bandage, after which the tourniquet was released.

Patients were transferred back to the ward immediately after recovery from anesthesia, and blood samples were collected promptly for coagulation function tests. All patients were instructed to perform active ankle pump exercises to prevent lower extremity venous thrombosis. Surgical drainage tubes were removed 24 h postoperatively, and elastic bandages were taken off at 72 h after surgery.

All enrolled patients underwent routine bilateral lower-extremity venous color Doppler ultrasound screening 1 day before surgery to identify preoperative isolated muscular calf vein thrombosis. A standardized follow-up ultrasound examination was uniformly scheduled on postoperative day 5 for every patient without exception. Emergency Doppler ultrasound was performed immediately for patients presenting with acute limb swelling, aggravated pain or elevated local skin temperature during hospitalization; meanwhile, the affected limb was immobilized and intermittent pneumatic compression was temporarily suspended. All licensed sonographers who performed ultrasound examinations were fully blinded to group allocation to avoid assessment bias. Predefined imaging criteria for complete thrombus resolution were as follows: full venous compressibility under compression ultrasound, complete disappearance of intraluminal abnormal echoes, and continuous unobstructed venous blood flow on color Doppler imaging. Partial thrombus regression or residual intraluminal echoes were defined as persistent venous thrombosis.

Each patient received daily subcutaneous injection of unfractionated heparin (0.4 mL, 4,100 IU), combined with 30-minute intermittent pneumatic compression of bilateral calves. Daily physical examinations of the lower limbs were conducted throughout hospitalization.

Once postoperative deep vein thrombosis (DVT) was confirmed, patients received once-daily subcutaneous enoxaparin sodium (low-molecular-weight heparin) at a dose of 150 IU/kg; inferior vena cava filters were implanted for patients with central venous thrombosis. Standardized oral rivaroxaban anticoagulation was administered after discharge. Patients without DVT received rivaroxaban 10 mg once daily for 3 weeks. For patients diagnosed with DVT, rivaroxaban 15 mg twice daily was prescribed for the initial 3 weeks, followed by a maintenance dose of 20 mg once daily thereafter.

Follow-up assessments were arranged at 1, 3 and 6 months after surgery, including measurement of knee flexion range of motion, documentation of postoperative complications, and Visual Analogue Scale (VAS) pain scoring. Anticoagulation therapy could only be discontinued when follow-up Doppler ultrasound confirmed complete thrombus resolution in accordance with the unified imaging criteria mentioned above. Long-term continuous anticoagulation was maintained if residual thrombus persisted without full resolution.

### Statistical methods

Statistical analysis was performed via SPSS 27.0. Continuous variables were expressed as mean ± SD or median (IQR); categorical data were presented as *n* (%). Intergroup comparisons were carried out by independent t-test/Mann–Whitney U test or Chi-square/Fisher's exact test, with mean difference, Cohen's d (for continuous variables) and OR (for binary variables) accompanied by 95% CI reported as effect sizes. Univariate binary logistic regression screened factors with *P* < 0.1 in univariate comparisons, and all significant variables were included in multivariate logistic regression to identify independent risk factors for postoperative DVT. Adjusted OR and 95% CI were reported for multivariate results. A two-tailed *P* < 0.05 indicated statistical significance.

## Results

### Patient characteristics and perioperative data

A total of 242 eligible patients were enrolled and stratified into two cohorts: the Study Group (with preoperative IMVT, *n* = 17) and the Comparison Group (without IMVT, *n* = 225). The baseline and perioperative characteristics are summarized in Table 2. No significant differences were observed between the two groups regarding the time from injury to surgery or operative time (*p* > 0.05), indicating comparability in these aspects. However, the Study Group demonstrated a significantly higher estimated intraoperative blood loss compared to the Comparison Group (143.5 ± 113.4 mL vs. 71.4 ± 77.5 mL, *p* < 0.001). The rate of intraoperative blood transfusion did not differ significantly between the groups.

**Table 2:** Operative characteristics and intraoperative outcomes.

Characteristic	Comparison Group	Study Group	Mean difference (95%CI)	Effect size (95%CI)	*p*-value
Time from injury to surgery (days),Mean(SD)	7.0 (4.8)	8.5 (3.5)	−1.47 [−3.38, 0.44]	Cohen's d = 4.77 [−0.80, 0.19]	0.221
Operative time (minutes),Mean(SD)	127.0 (66.2)	150.0 (54.1)	−22.96 [−55.40, 9.49]	Cohen's d = 65.48 [−0.84, 0.14]	0.165
Estimated blood loss (mL),Mean(SD)	71.4 (77.5)	143.5 (113.4)	−72.13 [−111.94, −32.32]	Cohen's d = 80.35 [−1.40, −0.40]	＜0.001
Intraoperative blood transfusion, n (%)	14 (6.2)	3 (17.6)		3.23 (95%CI:0.83∼12.57)	0.106

### Postoperative functional outcomes

The postoperative functional recovery and complications are detailed in Table 3. At the 1-month follow-up, the Study Group reported significantly higher VAS pain scores (4.7 ± 0.8 vs. 3.9 ± 1.1, *p* = 0.004) and exhibited a significantly lower mean knee flexion angle (67.9° ± 6.9° vs. 74.5° ± 8.9°, *p* = 0.004) compared to the Comparison Group. However, these differences were transient. By the 3-month follow-up, both pain scores and knee flexion angles were comparable between the two groups, with no significant differences observed at 3 and 6 months (*p* > 0.05). The swelling rate at discharge was similar between the groups.

**Table 3:** Postoperative outcomes and follow-up evaluations.

Outcome Measure	Comparison Group	Study Group	Mean difference (95%CI)	Effect size (95%CI)	*p*-value
VAS Pain Score (points)					
1 month，Mean(SD)	3.9（1.1）	4.7（0.8）	−0.78[−13.27, −2.33]	Cohen's d = 1.08[−1.22, −0.24]	0.004
3 months，Mean(SD)	1.4（0.7）	1.7（0.6）	−0.31[−0.66, 0.04]	Cohen's d = 0.71[−0.93, 0.059]	0.084
Knee flexion angle (°)					
1 month，Mean(SD)	74.5（8.9）	67.9（6.9）	6.52[2.15, 10.88]	Cohen's d = 8.81[0.24, 1.24]	0.004
3 months，Mean(SD)	109.4（6.8）	109.2（6.0）	0.14[−3.20, 3.47]	Cohen's d = 6.73[−0.47, 0.51]	0.935
6 months，Mean(SD)	130.2（6.5）	130.4（5.0）	−0.19[−3.37, 2.99]	Cohen's d = 6.42[−0.52, 0.46]	0.907
Swelling rate at discharge (%),Mean(SD)	6.7 (2.7)	7.2 (2.2)	−0.56[−1.88, 0.77]	Cohen's d = 2.67[−0.70, 0.29]	0.410
Postoperative DVT, n (%)	32	12		14.48 (95%CI:4.78∼43.85)	＜0.001

### Postoperative thrombotic complications

The incidence of postoperative deep vein thrombosis (DVT) was markedly higher in the study group (12/17, 70.6%) than in the comparison group (32/225, 14.2%), with a statistically significant difference (*p* < 0.001). Among patients who developed postoperative DVT, the proportion of non-isolated muscular calf vein thrombosis (proximal deep vein thrombosis involving the popliteal or femoral veins) was comparable between the two groups, accounting for 58.3% (7/12) in the study group and 59.4% (19/32) in the comparison group.

### Outcomes of postoperative DVT

At the one-month follow-up, DVT persisted in 25% (3/12) of the patients in the Study Group (with preoperative IMVT) and in 21.88% (7/32) of those in the Comparison Group (without preoperative IMVT), with no statistically significant difference between the groups (*p* > 0.05). By the three-month follow-up, all thrombi had resolved in the Comparison Group. However, two patients in the Study Group still exhibited persistent isolated muscular calf vein thrombosis.

### Consults

To clarify the clinical influence of preoperative isolated muscular calf vein thrombosis (IMVT) on perioperative safety and early postoperative prognosis following open reduction and internal fixation (ORIF) for Schatzker type II and III tibial plateau fractures, we strictly screened enrolled patients and excluded individuals with conventional high-risk factors for venous thrombosis, including malignant tumors, antiphospholipid syndrome, long-term oral contraceptive use, and personal or familial history of venous thromboembolism (17, 18). After rigorous screening, a total of 242 patients were finally included in this retrospective cohort analysis.

The present study demonstrated that patients with preoperative IMVT exhibited a significantly higher incidence of postoperative deep vein thrombosis (DVT) than those without IMVT, which was further verified as an independent risk factor through multivariate logistic regression analysis (OR=9.020, *p* < 0.001; Table 4). Notably, although the overall postoperative DVT rate differed substantially between groups, the anatomical distribution of thrombus subtypes was comparable among patients who developed postoperative DVT. The proportion of non-muscular proximal venous thrombosis was similar in the IMVT group and the comparison group, indicating that preoperative IMVT primarily increases the overall thrombotic risk rather than altering the anatomical pattern of secondary postoperative thrombosis.

**Table 4:** Multivariate binary logistic regression analysis for predictors of postoperative deep vein thrombosis.

Variables	OR	95%CI	*p*-value
Diabetes Mellitus	1.331	0.338∼5.235	0.682
Estimated blood loss	1.135	0.973∼1.361	0.131
1-month postoperative VAS score	1.407	0.759∼2.607	0.278
1-month postoperative knee flexion angle	0.919	0.850∼0.993	0.033
Postoperative DVT	9.020	2.461∼33.060	<0.001

In terms of perioperative surgical indicators, the IMVT group presented significantly greater intraoperative blood loss. This phenomenon can be reasonably explained by the unique local pathophysiological state induced by preoperative IMVT. Baseline muscular vein thrombosis may cause chronic venous stasis, increased capillary permeability, and aggravated preoperative soft tissue edema around the fractured knee. Such tissue congestion increases intraoperative vascular oozing during soft tissue dissection and fracture reduction, thereby elevating total intraoperative blood loss. More importantly, increased intraoperative bleeding may further exacerbate perioperative hypercoagulability via hemoconcentration and elevated blood viscosity. This prothrombotic state may synergize with preexisting IMVT, forming a combined prothrombotic mechanism that further increases the susceptibility to postoperative DVT (19). Therefore, increased intraoperative blood loss is not merely a concomitant clinical manifestation of preoperative IMVT but also serves as an intermediate adverse factor that amplifies postoperative thrombotic risk.

In addition to thrombotic outcomes, this study further analyzed early postoperative functional recovery. No significant intergroup difference was observed in preoperative VAS pain scores, indicating comparable baseline pain severity between the two cohorts. However, the IMVT group showed significantly higher pain scores and poorer knee flexion function at the 1-month postoperative follow-up, which was also independently correlated with postoperative thrombotic events according to regression results. These functional disparities gradually diminished over time, and no significant differences in pain level or knee range of motion were identified at 3 and 6 months postoperatively. These findings suggest that preoperative IMVT primarily affects early-stage postoperative functional recovery without interfering with long-term knee functional prognosis.

In terms of perioperative safety, no significant difference in overall complication rates was found between the two groups. No adverse events occurred in the IMVT group. In the comparison group, one patient developed acute pulmonary embolism and two patients suffered from joint stiffness, all of whom achieved favorable recovery after standardized intervention. Although preoperative IMVT significantly increases postoperative DVT risk, it does not appear to elevate the incidence of severe non-thrombotic perioperative complications.

Thrombus follow-up outcomes further reflected the adverse impact of preoperative IMVT. In patients without baseline IMVT, all postoperative thrombi achieved complete recanalization within 3 months. In contrast, several patients in the IMVT group presented persistent residual calf vein thrombosis at the early follow-up stage, indicating that preoperative IMVT may delay postoperative thrombus resolution and prolong the thrombotic recovery cycle.

### Study limitations

Several limitations of this study should be acknowledged. First, this was a single-center retrospective study, which inevitably carries inherent selection bias and residual confounding. Although we performed multivariate logistic regression to adjust for significant univariate variables, unmeasured factors, such as the exact severity of soft tissue injury and postoperative rehabilitation compliance, could not be fully balanced, limiting the strength of causal inference.

Second, the sample size was markedly imbalanced between groups. Preoperative IMVT combined with Schatzker II/III tibial plateau fractures is a relatively rare clinical condition. After screening nearly four consecutive years of inpatient data, only 17 eligible IMVT cases were enrolled. The imbalanced grouping was determined by the actual clinical incidence rather than artificial intervention, which may reduce statistical power and introduce potential type II error. The relatively small sample size of the IMVT group may also restrict the generalizability of the conclusions.

Third, although we adopted unified perioperative anticoagulation protocols and standardized ultrasound screening time points, all Doppler ultrasound examinations were operator-dependent. Despite sonographer blinding and unified imaging diagnostic criteria, minor measurement bias in thrombus detection and resolution evaluation could not be completely avoided.

Fourth, although multivariate adjustment was performed, retrospective statistical correction cannot eliminate all residual confounding factors. Further large-sample, prospective, controlled studies are required to validate our findings.

In conclusion, preoperative IMVT is independently associated with elevated postoperative DVT risk and impaired early knee functional recovery in patients undergoing ORIF for tibial plateau fractures. Although long-term functional prognosis remains unaffected, preoperative IMVT should be recognized as an important perioperative risk factor requiring intensified preoperative assessment and individualized thromboprophylaxis strategies.

## Data Availability

The original contributions presented in the study are included in the article/Supplementary Material, further inquiries can be directed to the corresponding authors.
